# Supplementary material for: Genomic diversity of non-diarrheagenic fecal Escherichia coli from children in sub-Saharan Africa and south Asia and their relatedness to diarrheagenic E. coli
Source: Nat Commun. 2023 Mar 14;14:1400. doi: 10.1038/s41467-023-36337-y (PMC10011798; doi:10.1038/s41467-023-36337-y)
Supplement: Supplementary file 1 — Supplementary Information [file 41467_2023_36337_MOESM1_ESM.pdf]

Supplementary Information for:

Genomic diversity of non-diarrheagenic fecal *Escherichia coli* from children in sub-Saharan Africa and south Asia their relatedness to diarrheagenic *E. coli* pathotypes

Genomic similarity of GEMS non-DEC fecal isolates to notable diarrheagenic pathotype isolates

To highlight the level of genetic similarity between the GEMS non-DEC fecal isolates and previously described pathotype *E. coli* linked to diarrheal illness we compared isolates of the ST173 lineage. This lineage contains six previously described ETEC reference genomes [31] as well as a GEMS non-DEC fecal isolate from a control sample and a previously described ST-ETEC pathotype isolate from GEMS that was associated with diarrhea [17] (Fig. 3a, Supplementary Data Set 3). Detection of all protein-coding genes predicted for the chromosome and plasmids of the GEMS ST-ETEC genome demonstrated there were multiple phage regions of the GEMS ST-ETEC genome absent from the GEMS non-DEC fecal isolate, as well as a putative copper resistance region, and several T6SS genes (Supplementary Data Set 4). Of the two plasmids carried by the GEMS ST-ETEC isolate, the larger plasmid (p504838_108) encoding a CS21 pilus was identified in the GEMS non-DEC genomes. Plasmid p504838_88 encoding one of the pathotype-defining ETEC virulence factors, the heat-stable enterotoxin (ST), as well as additional virulence factors, colonization factor CFA/I and the autotransporter EatA, was absent from the GEMS non-DEC fecal isolate. Another GEMS non-DEC fecal isolate exhibited remarkable genomic similarity to the Shiga-toxigenic O104:H4 German outbreak isolate C227-11 [32] (Fig. 3b, Supplementary Data Set 4). This GEMS non-DEC fecal isolate had the same ST (ST678) as C227-11 [32] and EAEC reference genomes, but lacked the Shiga toxin-encoding phage, plasmid-encoded EAEC virulence factors, and several genomic regions of the O104 outbreak isolate [32].

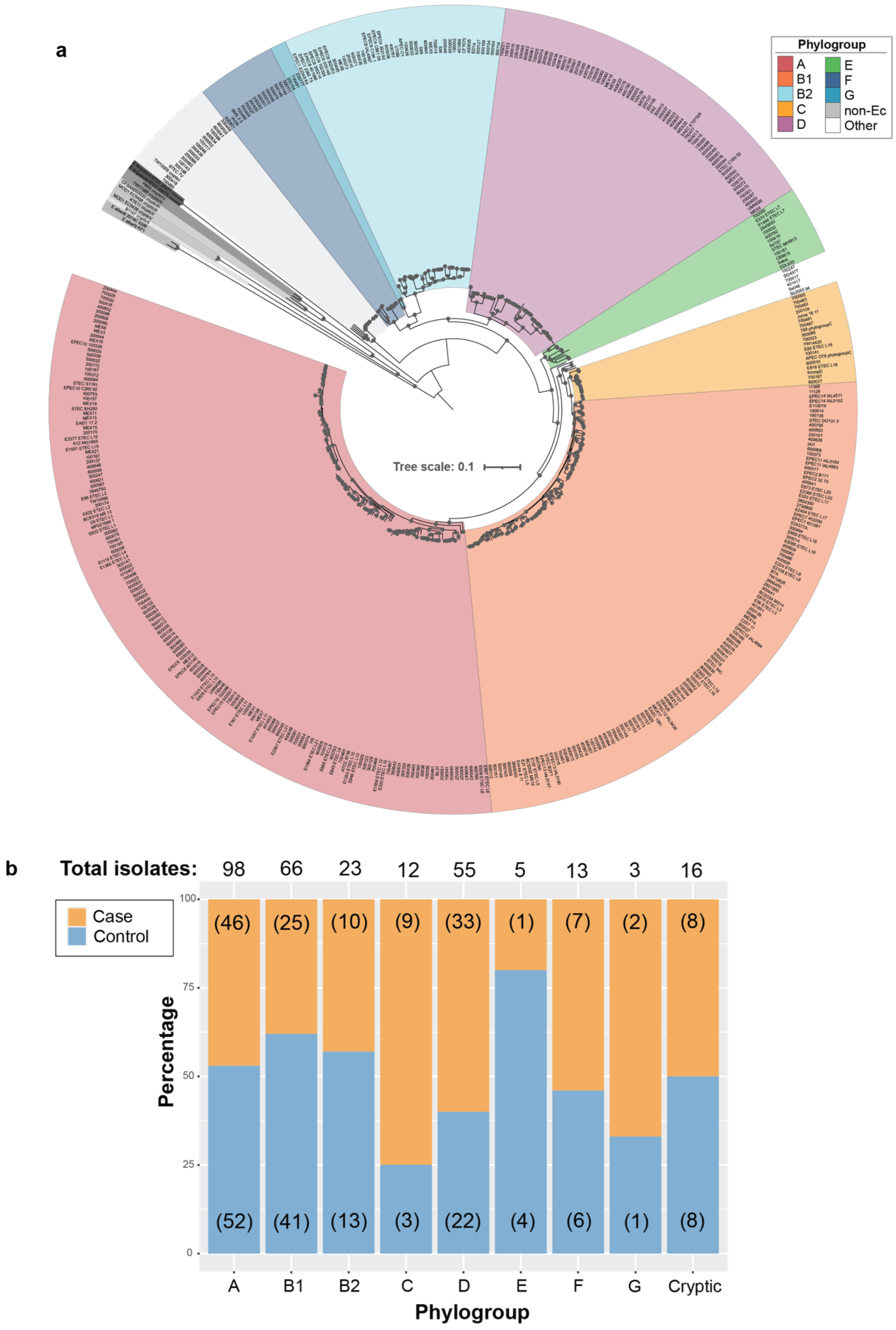
a
Phylogroup
A
B1
B2
C
D
E
F
G
non-Ec
Other
Tree scale: 0.1
b
Total isolates: 98 66 23 12 55 5 13 3 16
Case
Control
Percentage
100
75
50
25
0
(46) (25) (10) (9) (33) (1) (7) (2) (8)
(52) (41) (13) (3) (22) (4) (6) (1) (8)
A B1 B2 C D E F G Cryptic
Phylogroup

Supplementary Figure 1. Phylogenomic analysis with cryptic *Escherichia* clades and case vs. control comparison of phylogroup distribution. a) Phylogenomic analysis of the GEMS non-DEC fecal isolates with an expanded collection of reference genomes that include the representatives of the *Escherichia* cryptic lineages I to IV, as well as *E. albertii* and *E. fergusonnii* (Supplementary Data Set 1). The *E. coli* phylogroups are indicated by different colors (see inset figure legend) and the *Escherichia* cryptic clades, *E. albertii,* and *E. fergusonnii* are indicated by different shades of gray. Circles over each node indicate bootstrap support ≥95%. **b)** Numbers of GEMS non-DEC fecal isolates in each phylogroup that were isolated from case or control samples. The total number of GEMS non-DEC fecal isolates identified in each phylogroup is indicated at the top, and the number of case or control isolates are in parentheses. GEMS non-DEC fecal isolates from cases are indicated with orange and controls are indicated with blue. The counts of case and control genomes in each phylogroup are provided in Supplementary Data Set 2.

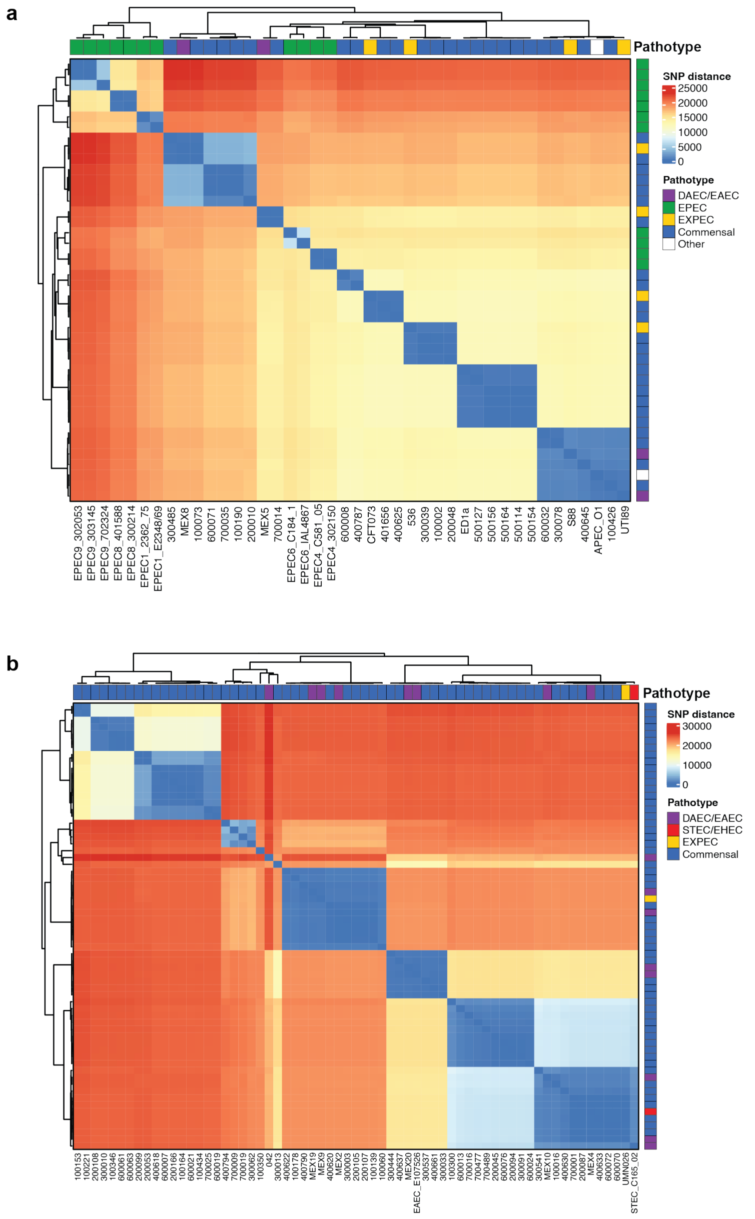

Supplementary Figure 2. Genomic similarity of GEMS non-DEC fecal isolates to pathotype *E. coli* of phylogroups B2 and D. Heat maps of the pairwise SNP distances among genomes of phylogroup B2 **(a)** and phylogroup D **(b)**. SNP distances were calculated from the core SNPs analyzed in the phylogenomic analysis of Figure 1. The row and column annotation of the heat maps indicates the pathotype association of each GEMS non-DEC genome or reference genome (see inset figure legend). The range of SNP distances and corresponding color scale is provided to the right of each heat map. The pairwise SNP distance matrix that served as input for this analysis is available in Supplementary Data Set 3.

Virulence Factors

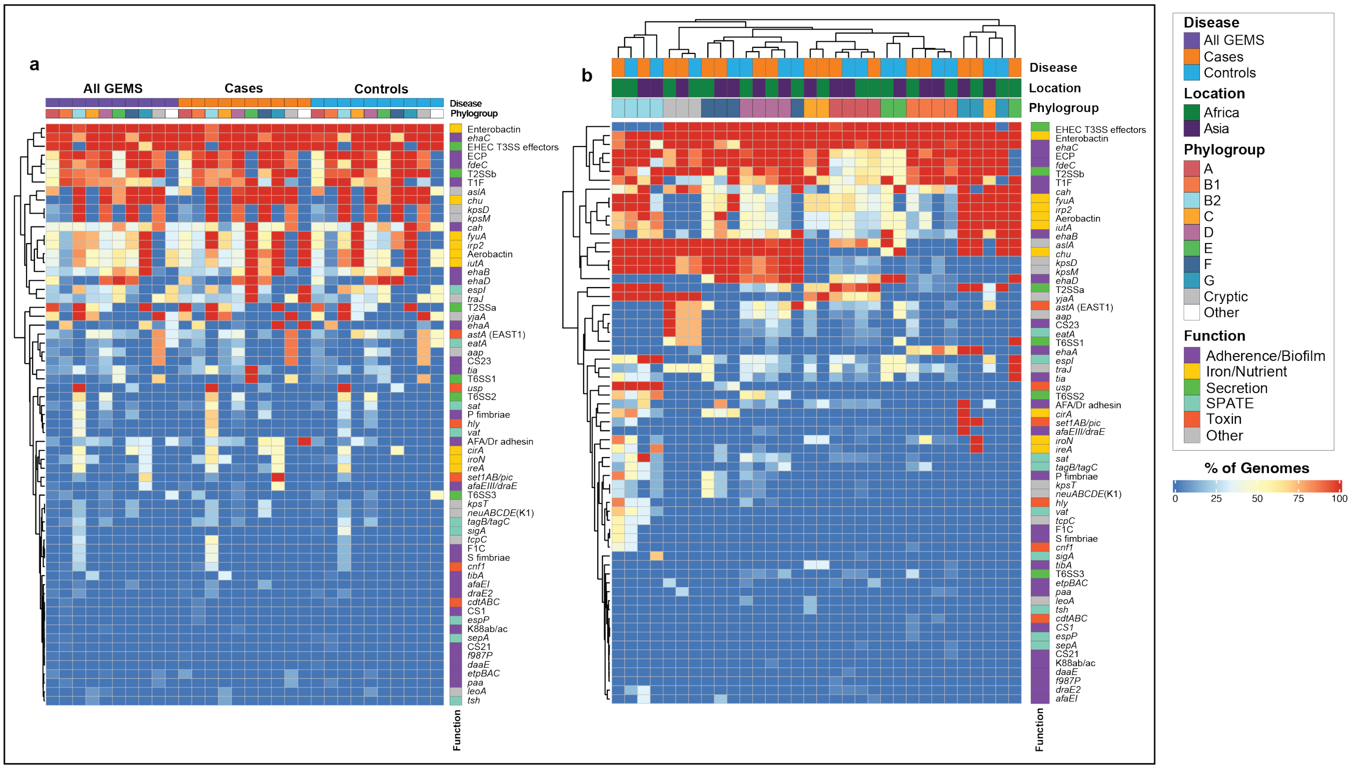

Antibiotic Resistance Genes

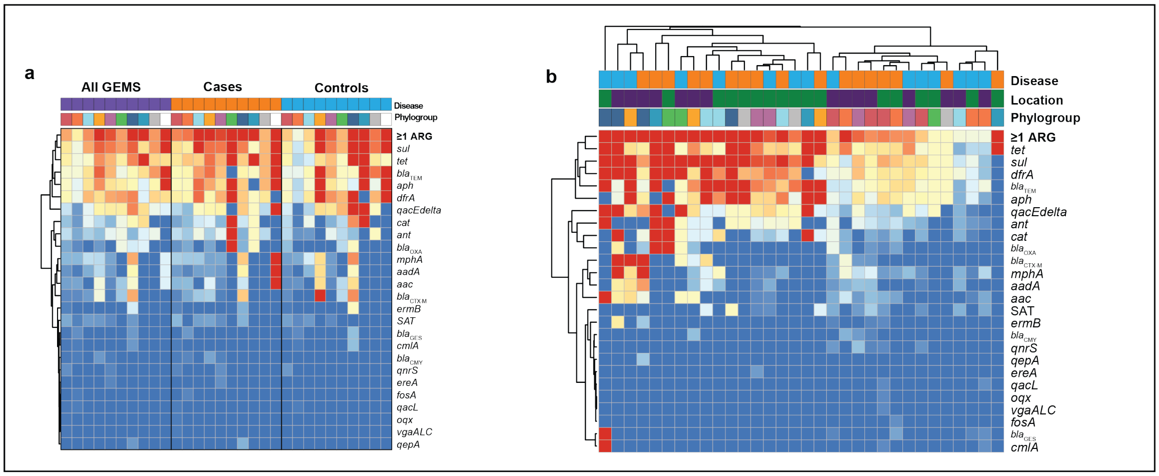

Plasmids

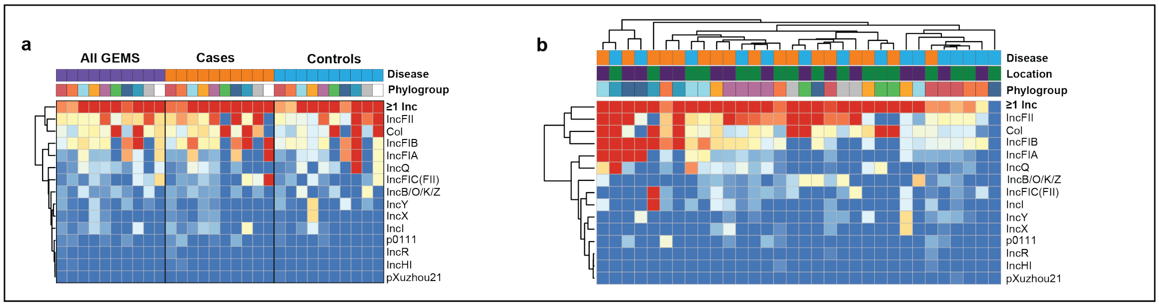

Supplementary Figure 3. Phylogroup associations of accessory virulence factors, antibiotic resistance genes, and plasmids among the GEMS non-DEC fecal isolates*.* The gene distribution was examined by phylogroup and disease association (case/control) **a)** considered overall and irrespective of geographic location, or **b)** compared by continent (Africa/Asia). The heat map indicates the percentage of genomes in each category (columns) that contain the virulence factors (VF), antibiotic resistance genes (ARGs), and plasmids (Inc; incompatibility types). The general functions of the accessory virulence factors are indicated by row annotation (see inset figure legend). The VF, ARG, and plasmid counts by phylogroup that served as input for this analysis is available in Supplementary Data Set 1.

Virulence Factors

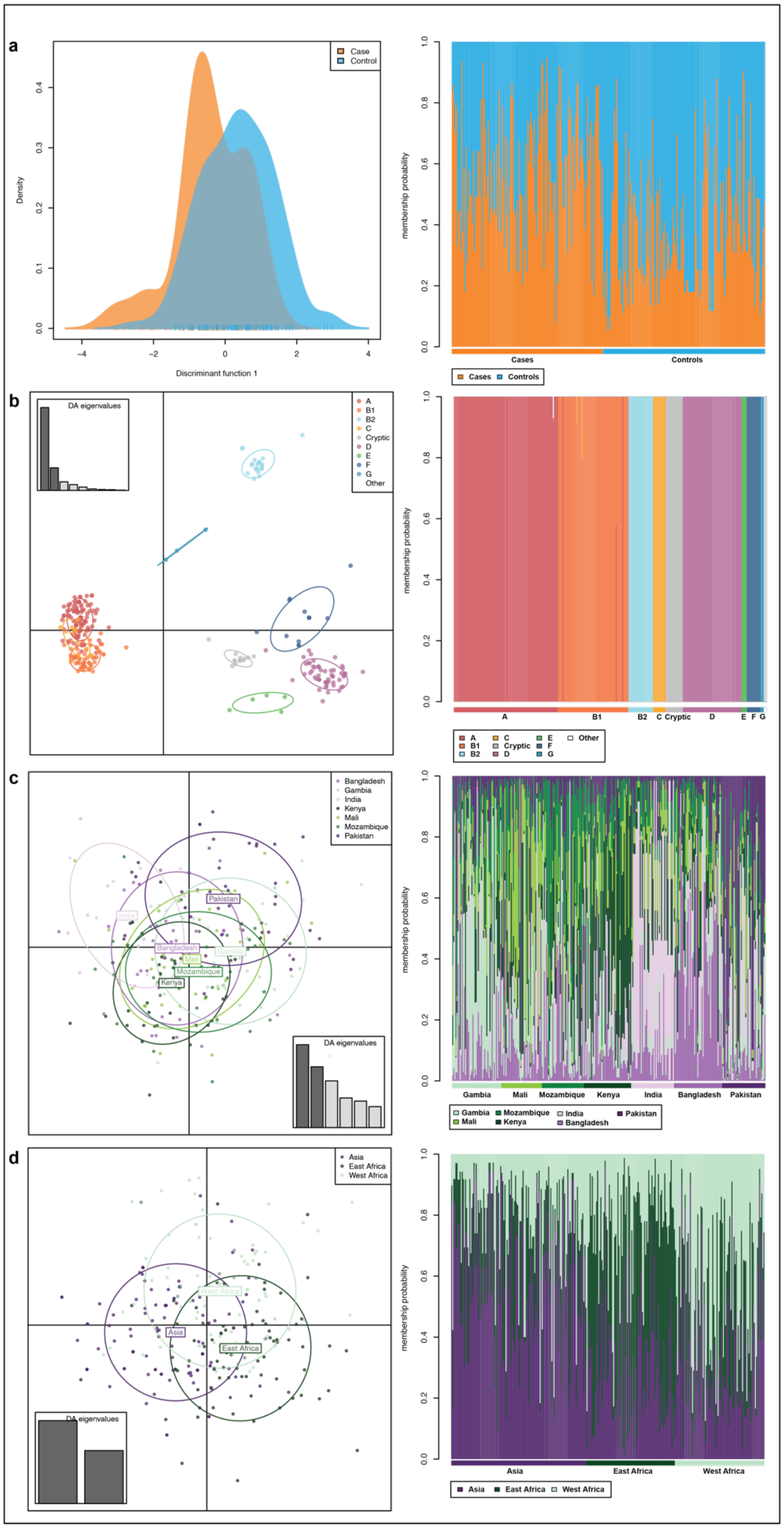

Antibiotic Resistance Genes (ARGs)

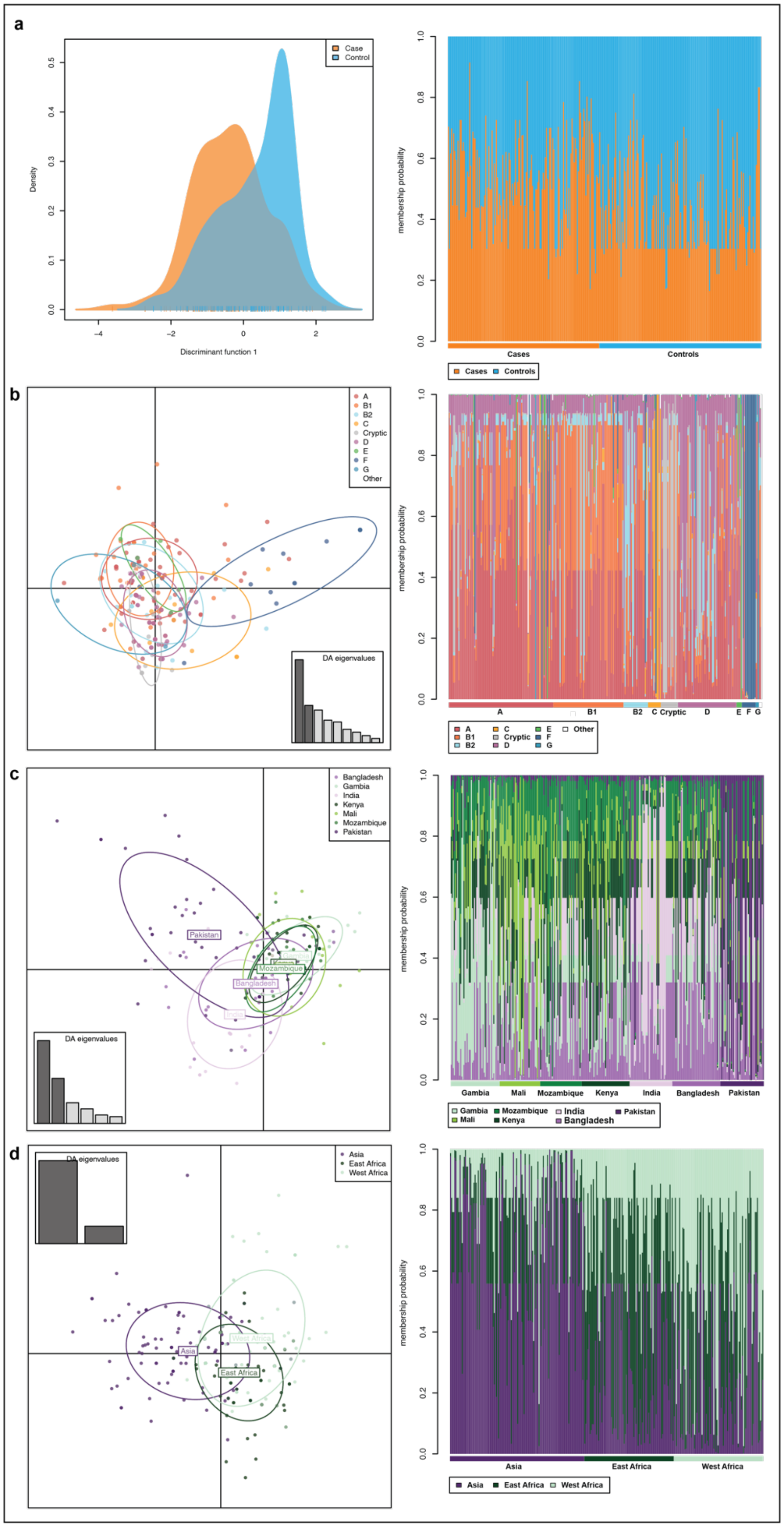

Supplementary Figure 4. Association of virulence factors and antibiotic resistance genes with diarrhea, phylogroup, GEMS site, and continent. Discriminant analysis of principal components (DAPC) was used to examine whether the virulence factors (VFs) and antibiotic resistance genes (ARGs) identified among the GEMS non-DEC fecal isolates exhibited associations with **a)** diarrhea (case vs. control), **b)** phylogroup, **c)** GEMS site, and **d)** continent. Each point in the scatter plots represents one of the GEMS genomes labeled by the pre-defined categories (disease, phylogroup, GEMS site, or continent) (see inset figure legends for color labels). A bar plot inset within each scatter plot shows the DA eigenvalues, which represent the ratio of the variance between groups over the ratio of the variance within groups for each of the DFs. The first two DFs are represented in each scatter plot and are indicated in dark gray in the bar plots. In the composition plots each genome is represented by a column with the color composition of each column representing the predicted group membership based on DAPC analysis. The genomes are ordered along the x-axis by their pre-defined group assignments, which is indicated by the bar and label beneath each plot. The VF and ARG matrices used as input for this analysis are available in Supplementary Data Set 1.

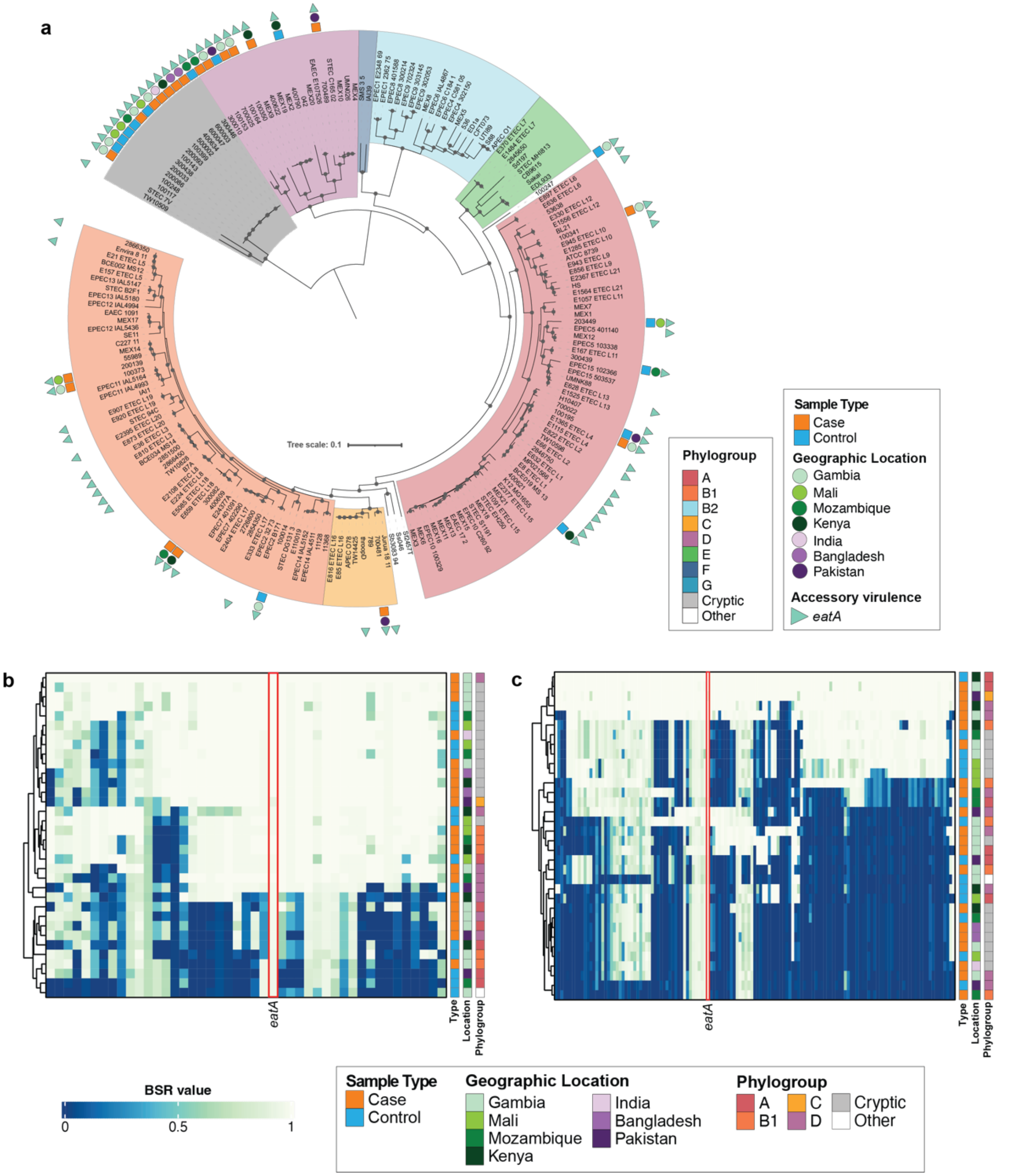

Supplementary Figure 5. Distribution and plasmid association of the virulence factor EatA among the GEMS non-DEC fecal isolates. a) Phylogenomic analysis of the *eatA*-containing GEMS non-DEC fecal isolates. Symbols on the outside of the phylogeny indicate the presence of the *eatA* gene (triangle), and the case/control sample association (square) and geographic location (GEMS sites; circle) of each of the isolates. **b)** Distribution of a partial *eatA*-containing IncFII plasmid (38,692 bp) from

GEMS non-DEC fecal isolate 100153. **c)** Distribution of a partial *eatA*-containing IncB/O/K/Z plasmid (117,360 bp) from GEMS non-DEC fecal isolate 400621 among the GEMS non-DEC fecal isolates that carry *eatA*. The BSR values close to one (light green) indicate gene presence, while values close to zero (dark blue) indicate gene absence. The plasmid detection data used as input for the heat maps is provided in Supplementary Data Set 5.

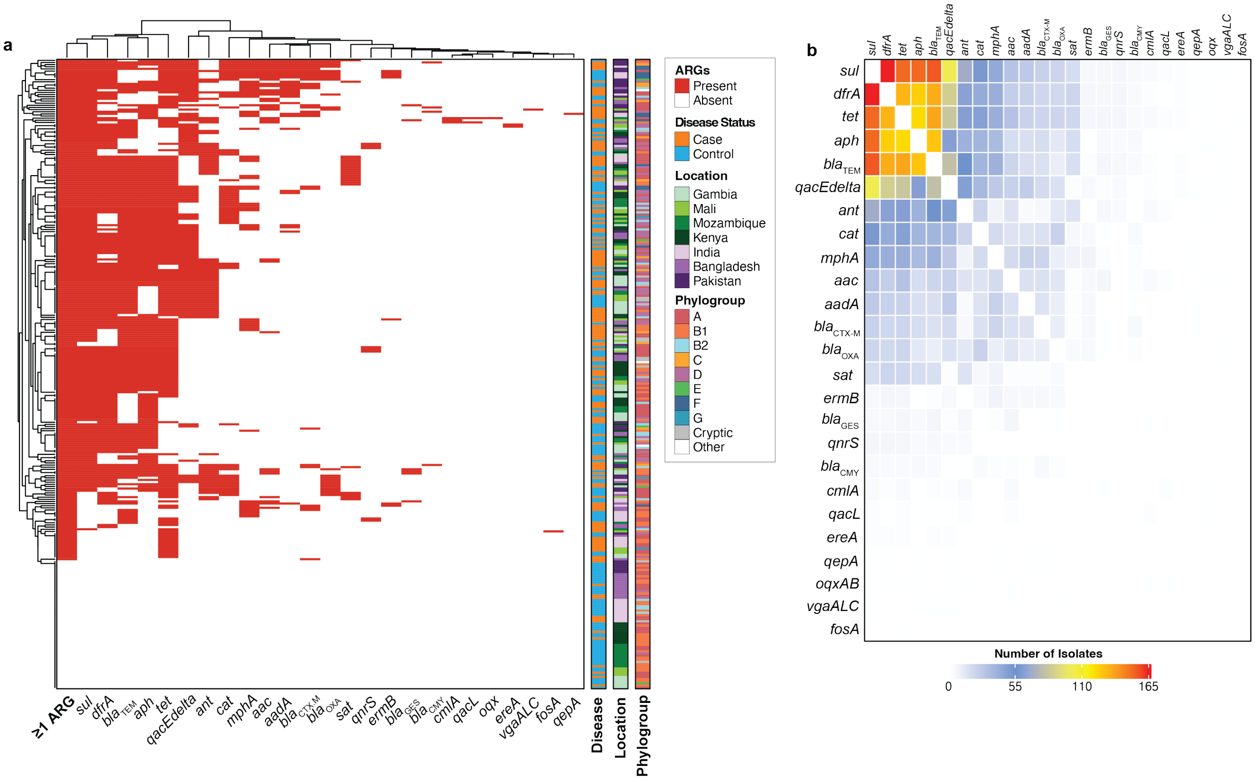

Supplementary Figure 6. Co-occurrence of antibiotic resistance genes (ARGs) in each of the GEMS non-DEC fecal isolates. a) The heat map indicates patterns of co-occurring ARGs among each of the 294 GEMS non-DEC genomes (rows). Row annotations indicate whether each isolate is from a case (diarrhea) or control (no diarrhea) sample, their geographic location of isolation, and phylogroup. **b)** Pairwise co-occurrence heat map indicating the total number of genomes that contain the different pairwise combinations of ARGs. The ARG matrices used as input for this analysis are available in Supplementary Data Set 1.
