# Supplementary material for: Genomic diversity of non-diarrheagenic fecal Escherichia coli from children in sub-Saharan Africa and south Asia and their relatedness to diarrheagenic E. coli
Source: Nat Commun. 2023 Mar 14;14:1400. doi: 10.1038/s41467-023-36337-y (PMC10011798; doi:10.1038/s41467-023-36337-y)
Supplement: Supplementary file 3 — Description of Additional Supplementary Information [file 41467_2023_36337_MOESM3_ESM.docx]

**Supplementary Information for:**

Genomic similarity of non-diarrheagenic fecal *Escherichia coli* from children in sub-Saharan Africa and south Asia to diarrheagenic *E. coli* pathotypes

**Supplementary Figures (**included in Supplementary Information**)**

**Supplementary** **Figure 1.** Phylogenomic analysis with cryptic *Escherichia* clades and case vs. control comparison of phylogroup distribution

**Supplementary** **Figure 2.** Genomic similarity of GEMS non-DEC fecal isolates to pathotype *E. coli* of phylogroups B2 and D.

**Supplementary** **Figure 3.** Phylogroup associations of accessory virulence factors, antibiotic resistance genes, and plasmids among the GEMS non-DEC fecal isolates*.*

**Supplementary** **Figure 4.** Association of virulence factors and antibiotic resistance genes with diarrhea, phylogroup, GEMS site, and continent.

**Supplementary** **Figure 5.** Distribution and plasmid association of the virulence factor EatA among the GEMS non-DEC fecal isolates*.*

**Supplementary** **Figure 6.** Co-occurrence of antibiotic resistance genes (ARGs) in each of the GEMS non-DEC fecal isolates.

**Supplementary Data Sets (**available for download separately**):**

**Supplementary** **Data Set 1.** Characteristics of the GEMS non-DEC fecal and *E. coli* reference genomes, and matrices indicating presence/absence of accessory virulence factors, antibiotic resistance genes, and plasmids.

**Supplementary** **Data Set 2.** Statistical analyses to examine associations of phylogroups, virulence factors, antibiotic resistance genes, and plasmids with diarrhea (case/control) or geographic location (continent and country).

**Supplementary** **Data Set 3.** SNP distances, patristic distances, and ST lineage assignments from the phylogenomic analysis in Figure 1.

**Supplementary** **Data Set 4.** Genomic comparisons of select closely related GEMS non-DEC fecal isolates and previously-described pathotype *E. coli*.

**Supplementary Data Set 5.** Distribution of two *eatA*-containing plasmids among the *eatA-*containing GEMS non-DEC fecal isolates*.*
