# Supplementary material for: Genomic diversity of non-diarrheagenic fecal Escherichia coli from children in sub-Saharan Africa and south Asia and their relatedness to diarrheagenic E. coli
Source: Nat Commun. 2023 Mar 14;14:1400. doi: 10.1038/s41467-023-36337-y (PMC10011798; doi:10.1038/s41467-023-36337-y)
Supplement: Supplementary file 9 — Reporting Summary [file 41467_2023_36337_MOESM9_ESM.pdf]

nature portfolio

Corresponding author(s): David Rasko, PhD

Last updated by author(s): Dec 16, 2022

Reporting Summary

Nature Portfolio wishes to improve the reproducibility of the work that we publish. This form provides structure for consistency and transparency in reporting. For further information on Nature Portfolio policies, see our Editorial Policies and the Editorial Policy Checklist.

Statistics

For all statistical analyses, confirm that the following items are present in the figure legend, table legend, main text, or Methods section.

n/a	Confirmed	
☐	☒	The exact sample size (n) for each experimental group/condition, given as a discrete number and unit of measurement
☒	☐	A statement on whether measurements were taken from distinct samples or whether the same sample was measured repeatedly
☐	☒	The statistical test(s) used AND whether they are one- or two-sided<br>*Only common tests should be described solely by name; describe more complex techniques in the Methods section.*
☒	☐	A description of all covariates tested
☒	☐	A description of any assumptions or corrections, such as tests of normality and adjustment for multiple comparisons
☒	☐	A full description of the statistical parameters including central tendency (e.g. means) or other basic estimates (e.g. regression coefficient) AND variation (e.g. standard deviation) or associated estimates of uncertainty (e.g. confidence intervals)
☐	☒	For null hypothesis testing, the test statistic (e.g. F, t, r) with confidence intervals, effect sizes, degrees of freedom and P value noted<br>*Give P values as exact values whenever suitable.*
☒	☐	For Bayesian analysis, information on the choice of priors and Markov chain Monte Carlo settings
☒	☐	For hierarchical and complex designs, identification of the appropriate level for tests and full reporting of outcomes
☒	☐	Estimates of effect sizes (e.g. Cohen's d, Pearson's r), indicating how they were calculated

Our web collection on statistics for biologists contains articles on many of the points above.

Software and code

Policy information about availability of computer code

Data collection	Data were collected and examined in Excel spreadsheets and R v.4.1.0
Data analysis	Trimmomatic v.0.36 (https://github.com/usadellab/Trimmomatic), SPAdes v.3.11.1 (https://github.com/ablab/spades), BIGSdb 1.32.1 (https://github.com/kjolley/BIGSdb/), BLAST (ftp://ftp.ncbi.nlm.nih.gov/blast/executables/blast+/LATEST/), SerotypeFinder v1.0.0 (https://github.com/tseemann/abricate/issues/40), Northern Arizona SNP Pipeline (NASPv1) (https://github.com/TGenNorth/NASP), IQ-TREE v.1.6.12 (https://github.com/iqtree), interactive tree of life (iTOL) v.5 (https://itol.embl.de), ggplot2 v.3.3.5 (https://github.com/ggplot2), R v.4.1.0 (https://www.r-project.org/), snp-dists 0.8.2 (https://github.com/tseemann/snp-dists), ComplexHeatmap v.2.8.0 (https://github.com/jokergoo/ComplexHeatmap), ape 5.5 (https://github.com/cran/ape), PlasmidFinder database v.2021-11-29 (https://anaconda.org/bioconda/plasmidfinder), resistance gene identifier (RGI) v.5.2.0 (https://github.com/arpcard/rgi), Circos v.0.69-9 (https://github.com/vigsterkr/circos), ergatis v.2 (https://github.com/jorvis/ergatis), RAST v.2.0 (https://rast.nmpdr.org/)<br>comprehensive antibiotic resistance database (CARD) v.3.1.4 (https://github.com/arpcard), BLAST score ratio (BSR) (https://github.com/jasonsahl/LS-BSR), virulence factor database (VFDB 2019) (https://github.com/haruosuz/vfdb), adegenet v. 2.1.4 (https://github.com/thibautjombart/adegenet)

For manuscripts utilizing custom algorithms or software that are central to the research but not yet described in published literature, software must be made available to editors and reviewers. We strongly encourage code deposition in a community repository (e.g. GitHub). See the Nature Portfolio guidelines for submitting code & software for further information.

Data

Policy information about availability of data

All manuscripts must include a data availability statement. This statement should provide the following information, where applicable:

- Accession codes, unique identifiers, or web links for publicly available datasets
- A description of any restrictions on data availability
- For clinical datasets or third party data, please ensure that the statement adheres to our policy

Data availability. All sequence data and genome assemblies generated in this study have been submitted to GenBank under the BioProject PRJNA611810. The individual assembly accession numbers and Illumina sequence read accession numbers are listed in Data Set S1.

Human research participants

Policy information about studies involving human research participants and Sex and Gender in Research.

Reporting on sex and gender	Not human subject research. All data for the GEMS participants and study are included in publicly available ClinEpiDB (https://clinepidb.org/ce/app).
Population characteristics	Not human subject research. All data for the GEMS participants and study are included in publicly available ClinEpiDB (https://clinepidb.org/ce/app).
Recruitment	Not human subject research. All data for the GEMS participants and study are included in publicly available ClinEpiDB (https://clinepidb.org/ce/app).
Ethics oversight	Not human subject research. All data for the GEMS participants and study are included in publicly available ClinEpiDB (https://clinepidb.org/ce/app).

Note that full information on the approval of the study protocol must also be provided in the manuscript.

Field-specific reporting

Please select the one below that is the best fit for your research. If you are not sure, read the appropriate sections before making your selection.

☐ Life sciences ☐ Behavioural & social sciences ☒ Ecological, evolutionary & environmental sciences

For a reference copy of the document with all sections, see nature.com/documents/nr-reporting-summary-flat.pdf

Ecological, evolutionary & environmental sciences study design

All studies must disclose on these points even when the disclosure is negative.

Study description	The goal of this study was to examine the genomic diversity of Escherichia coli isolate from children under the age of five in low and middle income countries examined in the Global Enteric Multicenter Study.
Research sample	The complete sample set of the GEMS isolates, collected from Dec 1, 2007, and March 3, 2011, were examined for E. coli isolates that lacked virulence factors that placed the isolates into known pathogenic groups. Isolates were selected from children that exhibited diarrhea (cases) and those that did not (controls).
Sampling strategy	The sample set consists of 350 isolates (25 diarrhea cases and 25 controls, from each of the seven GEMS sites) that were selected based on the lack of presence of the PCR positive test for key virulence factors of Escherichia coli. From this initial selection 336 were able to be recovered from the freezer or yielded appropriate genome metrics to be considered E. coli. Of those, the genomes of 42 isolates contained virulence factors that would have suggested they were pathogenic E. coli and were removed from further analysis.
Data collection	Data were collected and examined via excel spreadsheets and R-workbooks by THH, JM, SMT and DAR
Timing and spatial scale	The study period for the collection of the isolates was Dec 1, 2007, and March 3, 2011.
Data exclusions	The sample set consists of 350 isolates (25 diarrhea cases and 25 controls, from each of the seven GEMS sites) that were selected based on the lack of presence of the PCR positive test for key virulence factors of Escherichia coli. From this initial selection 336 were able to be recovered from the freezer or yielded appropriate genome metrics to be considered E. coli. Of those, the genomes of 42 isolates contained virulence factors that would have suggested they were pathogenic E. coli and were removed from further analysis.
Reproducibility	Analyses were completed at least twice to ensure rigor and all were successful
Randomization	Not applicable.

Blinding	Not applicable.

Did the study involve field work? ☐ Yes ☒ No

Reporting for specific materials, systems and methods

We require information from authors about some types of materials, experimental systems and methods used in many studies. Here, indicate whether each material, system or method listed is relevant to your study. If you are not sure if a list item applies to your research, read the appropriate section before selecting a response.

Materials & experimental systems

n/a	Involved in the study
☒	☐ Antibodies
☒	☐ Eukaryotic cell lines
☒	☐ Palaeontology and archaeology
☒	☐ Animals and other organisms
☒	☐ Clinical data
☒	☐ Dual use research of concern

Methods

n/a	Involved in the study
☒	☐ ChIP-seq
☒	☐ Flow cytometry
☒	☐ MRI-based neuroimaging
